# Increased oxidative stress associated with the severity of the liver disease in various forms of hepatitis B virus infection

**DOI:** 10.1186/1471-2334-5-95

**Published:** 2005-10-31

**Authors:** Cengiz Bolukbas, Fusun Filiz Bolukbas, Mehmet Horoz, Mehmet Aslan, Hakim Celik, Ozcan Erel

**Affiliations:** 1 Department of Internal Medicine, Gastroenterology Division, Harran University, Medical Faculty, Sanliurfa, Turkey; 2 Department of Internal Medicine, Harran University, Medical Faculty, Sanliurfa, Turkey; 3 Department of Biochemistry, Harran University, Medical Faculty, Sanliurfa, Turkey

## Abstract

**Background:**

Oxidative stress can be defined as an increase in oxidants and/or a decrease in antioxidant capacity. There is limited information about the oxidative status in subjects with hepatitis B virus infection. We aimed to evaluate the oxidative status in patients with various clinical forms of chronic hepatitis B infection.

**Methods:**

Seventy-six patients with hepatitis B virus infection, in whom 33 with chronic hepatitis, 31 inactive carriers and 12 with cirrhosis, and 16 healthy subjects were enrolled. Total antioxidant response and total peroxide level measurement, and calculation of oxidative stress index were performed in all participants.

**Results:**

Total antioxidant response was significantly lower in cirrhotics than inactive HbsAg carriers and controls (p = 0.008 and p = 0.008, respectively). Total peroxide level and oxidative stress index was significantly higher in cirrhotic (p < 0.001, both) and chronic hepatitis B subjects (p < 0.001, both) than inactive HbsAg carriers and controls. Total antioxidant response was comparable in chronic hepatitis B subjects, inactive HbsAg carriers and controls (both, p > 0.05/6). Total peroxide level and oxidative stress index were also comparable in inactive HBsAg carriers and controls (both, p > 0.05/6). Serum alanine amino transferase level was positively correlated with total peroxide level and oxidative stress index only in chronic hepatitis B subjects (p = 0.002, r = 0.519 and p = 0.008, r = 0.453, respectively).

**Conclusion:**

Oxidative stress occurs secondarily to increased total lipid peroxidation and inadequate total antioxidant response and is related to severity of the disease and replication status of virus in hepatitis B infection.

## Background

Reactive oxygen species (ROS) are oxygen-containing molecules that produced during normal metabolism [1]. The organism has enzymatic and non-enzymatic antioxidant systems neutralizing the harmful effects of the endogenous ROS products [2]. Under certain conditions, the oxidative or anti-oxidative balance shifts towards the oxidative status as a result of increase in ROS and/or impairment in antioxidant mechanism [3,4].

It has been suggested that reactive oxygen species and lipid peroxidation products likely contribute to both onset and progression of hepatic fibrosis [5]. In addition, oxidative stress is one of the reasons of DNA damage, which might be associated with the development of hepatocellular carcinoma (HCC) in chronic viral hepatitis [6].

In several studies [7-10], increased oxidative stress has been suggested to be responsible from the hepatocellular damage caused by chronic hepatitis B infection (CHB). However, most of them have evaluated oxidative status using individual antioxidants measurement and the information about the total antioxidant response (TAR) of subjects with CHB and cirrhosis due to hepatitis B virus (HBV) infection is limited [11]. To our knowledge, there is no information in the literature about the oxidants in subjects with cirrhosis due to HBV infection, and neither oxidants nor antioxidants in inactive hepatitis B carriers.

In the present study, we aimed to measure the TAR in CHB, cirrhosis due to HBV infection and inactive HbsAg carrier subjects to evaluate their antioxidant status using a novel automated method [12]. As a reciprocal measure, the total peroxide levels of the same plasma samples were also measured. The percent ratio of the total plasma peroxide level to the plasma TAR value was regarded as oxidative stress index [13].

## Methods

### Enrollment of patients

CHB subjects (n = 33, 20 male/13 female; mean age; 39 ± 12.8 years), inactive HBsAg carrier subjects (n = 31, 19 male/12 female; mean age; 38 ± 8.3 years), subjects with cirrhosis due to HBV infection (n = 12, 7 male/5 female; mean age 40.9 ± 11.6 years) and healthy controls (n = 16, 9 male/7 female; mean age 31.6 ± 6 years) were enrolled in the present study. Patient selection has been started at March 2003 and finished at December 2004, when the numbers of study subjects have reached to provide a power for statistical analysis. Sample size was calculated with an expected parameter estimate based on a pilot study performed in our department. An assuming a mean of 1.71 TAR in control group and a mean of 1.40 TAR in Cirrhosis-HBV Group with a 0.25 standard deviation, the minimum sample size thus required to be approximately 13 in each study groups within a 95% confidence and 80% power.

All cirrhotic subjects had compensated and Child A cirrhosis according to the Child-Pugh classification. All participants were age and sex matched. The study protocol was carried out in accordance with the Helsinki Declaration as revised in 1989. All subjects were informed about the study protocol and the written consent was obtained from each one.

### Exclusion criteria

Exclusion criteria included the use of supplemental vitamins, serum total bilirubin level higher than 2 mg/dL, history of diabetes mellitus, coronary artery disease, rheumatoid arthritis, cancer, systemic or local infection, the existence of alcohol intake, poor nutritional status, pregnancy, decompensated and Child B or C cirrhosis, concomitant chronic hepatitis C (CHC) or hepatitis D or other well known liver diseases such as metabolic or autoimmune disorders and various infectious states of the liver, non-alcoholic steatohepatitis and HBV-DNA negativity in patients with CHB and cirrhosis.

HCC was detected using ultrasonographic examination of the liver and measurement of serum alpha fetoprotein level. The ultrasound and AFP determinations were performed before the enrolment and especially before drawing the blood samples in all subjects.

### Initial evaluation

Diagnosis of chronic hepatitis B was based on 6 months history of HBsAg and HBV-DNA positivity with at least 2 times higher alanine amino transferase (ALT) than upper limit of normal level. Findings of chronic hepatitis B were supported by the histopathological evaluation based on the modified Knodell score [14]. Inactive HBsAg carrier state for HBV infection was diagnosed on the basis of at least 1 year of HBsAg positivity with normal ALT levels and negative HBV-DNA.

Cirrhosis due to HBV was diagnosed on the basis of the clinical, laboratory, virological, radiological, and/or histopathological findings.

Control group consisted of healthy individuals with normal medical history, physical examination, blood biochemistry and negative hepatitis C virus (HCV) antibody and HBV serum markers.

A wide panel of biochemical and haematological parameters were evaluated by standard automated techniques.

### Virological studies

Anti-HCV, HBsAg, anti-HBs, HBeAg, anti-Hbe were assayed by micro particle enzyme immunoassay (MEIA) (Abbott axsym system, IL USA). The presence of hepatitis D infection was detected by enzyme immunoassay for detection of antibodies against hepatitis delta [(Abbott Murex, Dartford UK) (organon Teknika)]. HBV-DNA was investigated using real time polymerase chain reaction (PCR) method [HBV QNP 2.0 HBV-DNA quantitative kits, Iontek, Istanbul, Turkey) (BioRad ÝCycler)]. Upper and lower limit of HBV-DNA level with real time PCR were 2 × 10^2 ^and 2 × 10^7 ^copy/ml, respectively.

### Blood collection

Blood samples were obtained following an overnight fasting state. Smoker subjects were not permit to smoke during those fasting period. Samples were withdrawn from a cubital vein into heparinised tubes and immediately stored on ice at 4°C. The plasma was then separated from the cells by centrifugation at 3000 rpm for 10 min. Because we aimed to analyze all plasma samples simultaneously for the measurement of TAR and total peroxide level, and the collection of the samples were thought to be longer than 1 month of period, plasma samples were stored at -80°C until analysis as described elsewhere [15,16].

### Measurement of the total antioxidant status of plasma

The total antioxidant status of the plasma was measured using a novel automated colorimetric measurement method for TAR developed by Erel [12]. In this method the hydroxyl radical, the most potent biological radical, is produced by the Fenton reaction, and reacts with the colourless substrate O-dianisidine to produce the dianisyl radical, which is bright yellowish-brown in colour. Upon the addition of a plasma sample, the oxidative reactions initiated by the hydroxyl radicals present in the reaction mix are suppressed by the antioxidant components of the plasma, preventing the colour change and thereby providing an effective measure of the total antioxidant capacity of the plasma. The assay results are expressed as mmol Trolox eq./L, and the precision of this assay is excellent, being lower than 3% [17].

### Measurement of total plasma peroxide concentration

The total plasma peroxide concentrations were determined using the FOX2 method [18] with minor modifications [13]. The FOX2 test systemis based on the oxidation of ferrous iron to ferric iron by the various types of peroxides contained in the plasma samples, in the presence of xylenol orange which produces a coloured ferric-xylenol orange complex whose absorbance can bemeasured. TheFOX2 reagent was prepared by dissolving ammonium ferrous sulphate (9.8 mg) in 250 mM H2SO4 (10 ml) to give a final concentration of 250 mM ferrous iron in acid. This solution was then added to 90 ml HPLC-grade methanol containing 79.2 mg butylated hydroxytoluene (BHT). Finally, 7.6 mg xylenol orange was added, with stirring, to make the working reagent (250 mM ammonium ferrous sulphate, 100 mMxylenol orange, 25 mM H2SO4, and 4 nM BHT, in 90% (v/v) methanol in a final volume of 100 ml). The blank reagent contained all the components of the solution except ferrous sulphate.

Aliquots (200 mL) of plasma were mixed with 1.8 ml FOX2 reagent. After incubation at room temparature for 30 min, the vials were centrifuged at 12,000 g for 10 min. The absorbance of the supernatant was then determined at 560 nm. The total peroxide content of the plasma samples was determined as a function of the difference in absorbance between the test and blank samples using a solution of H2O2 as standard. The coefficient of variation for individual plasma samples was less than 5%.

### Oxidative stress index

The percent ratio of the total peroxide to the total anti-oxidant potential gave the oxidative stress index, an indicator of the degree of oxidative stress [13].

### Statistical analysis

Continuous variables were compared by Kruskal-Wallis one-way analysis of variance for non-parametric data with a post hoc analysis using a Mann-Whitney U test. Parametric variables were compared using One-way analysis of variance with post hoc analysis using Tukey test. Fisher's exact test was used to test the sex differences between groups. Spearman's correlation analysis was used to find out the relationship of alanine aminotransferase with TAR, total peroxide level or OSI. Data were presented as median and range for nonparametric variables and mean ± SD for parametric variables. Differences were regarded as significant at 0.05/6 in Kruskal-Wallis one-way analysis and p < 0.05 in other analysis.

## Results

Mean age, gender distribution were equal in each group. Serum ALT levels were higher in both CHB and cirrhotic subjects than controls (p = 0.001, p = 0.032, respectively). While CHB subjects had higher serum ALT levels than inactive HBsAg carriers (p = 0.001), both cirrhotics and control groups did not show any significant difference in term of serum ALT levels with inactive HBsAg carriers. There was no statistically significant difference in respect to percent of smokers, numbers of cigarettes smoking in a day and smoking duration of the groups (all p > 0.05). All subjects who smoked were smoking filtered cigarettes.

Smoking habit details and other clinical and demographic data are shown in Table 1.

**Table 1:** The clinical and demographic data of the study groups

	**CHB Group**	**Inactive HBsAg Carrier Group**	**Cirrhosis-HBV Group**	**Control Group**
**n**	33	31	12	16
**Age (years)**	39 ± 12.8	38 ± 8.3	40.9 ± 11.6	31.6 ± 6
**Gender (M/F)**	20/13	19/12	7/5	9/7
**ALT IU/L**	100 ± 32*	24.8 ± 9.3	40.6 ± 18.5**	17.5 ± 8.4
**Smoking Habit**				
**Percent**	45.45	41.93	41.66	43.75
**Cigarettes/day**	8.8 ± 4.1	7.8 ± 3.1	9.2 ± 3.8	9.8 ± 3.1
**Duration(years)**	15.5 ± 9.4	16.6 ± 7.0	14.4 ± 8.9	12 ± 5.9

Data were presented as mean ± SD.

* p < 0.001 CHB group vs. inactive HBsAg carriers and control.

** p < 0.05 HBV related cirrhosis group vs. control.

Qualitative variables were assessed by Fisher's exact tests. Differences in continuous variables were evaluated by One-way analysis of variance with post hoc analysis using Tukey test. A p value of < 0.05 was considered statistically significant.

CHB, chronic hepatitis B; HBsAg, hepatitis B surface antigen; HBV, hepatitis B virus; ALT, alanine aminotransferase.

TAR was significantly lower in cirrhotic subjects than the inactive HBsAg carrier and controls (p = 0.008 and p = 0.008, respectively). The difference between subjects with CHB and cirrhotic subjects in respect to TAR was not statistically significant (p > 0.05/6). There was no significant difference in TAR between chronic hepatitis B and inactive HBsAg carrier or controls (all p > 0.05/6). (Table 2)

**Table 2:** Oxidative and antioxidative parameters in each group.

	**CHB Group**	**Inactive HBsAg Carrier Group**	**Cirrhosis-HBV Group**	**Control Group**
**TAR (mmol Trolox eq./L)**	1.58 (1.16–2.23)	1.62 (1.3–1.98)	1.36 (1.2–1.9)‡	1.7 (1.46–1.96)
**Total peroxide (μmol H_2_O_2_/L)**	32.2(18.9–66)*	25.9(6.6–32)	38.5(22.5–59)**	24.8(18.5–31)
**OSI (AU)**	1.9 (1–3.9)*	1.6 (0.4–2.4)	2.66(1.1–4.9)**	1.4 (1–1.8)

Data were presented as median and range.

‡ p < 0.008 HBV related cirrhosis vs. inactive HBsAg carriers and control.

* p < 0.001 CHB vs. inactive HBsAg carriers and control.

** p < 0.001 HBV related cirrhosis vs. inactive HBsAg carriers and control.

Differences were regarded as significant at 0.05/4 in Kruskal-Wallis one-way analysis.

CHB, chronic hepatitis B; HBsAg, hepatitis B surface antigen; HBV, hepatitis B virus; TAR, total antioxidant response; OSI, oxidative stress index.

Total plasma peroxide level of CHB or cirrhotic subjects was significantly higher than inactive HBsAg carrier and controls (p < 0.001, p = 0.001 and p < 0.001, p = 0.001, respectively). The total plasma peroxide level and OSI, an indicator of the degree of oxidative stress, were not significantly higher in cirrhotic than chronic hepatitis B subjects (all p > 0.05/6). (Table 2)

OSI was significantly higher in CHB and cirrhotic subjects than inactive HBsAg carriers and controls (p < 0.001, p < 0.001 and p < 0.001, p < 0.001 respectively) (Table 2).

Inactive HbsAg carriers and controls had comparable results in term of TAR, total peroxide level and OSI (all p > 0.05/6).

**Figure 1:**
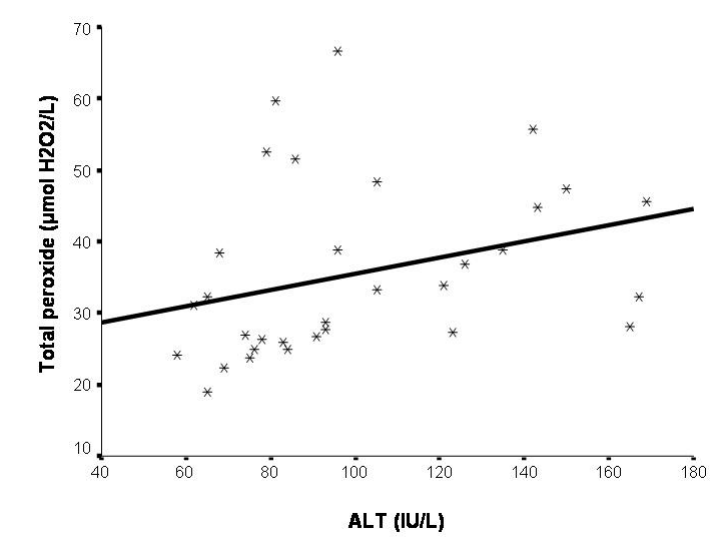
Serum ALT values were positively correlated with total peroxide levels in subjects with chronic hepatitis B (p = 0.002, r = 0.519). ALT, alanine amino transferase.

**Figure 2:**
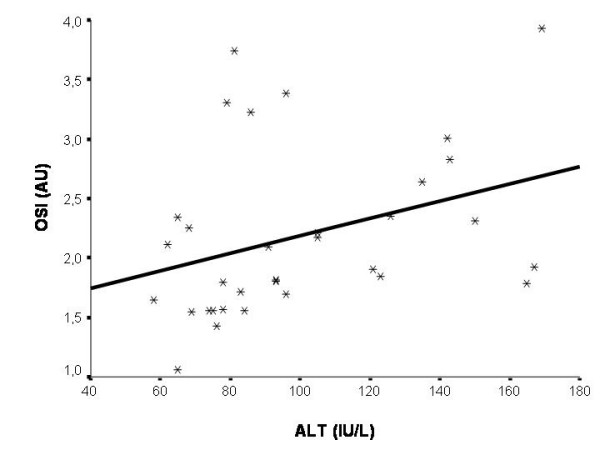
Serum ALT values were positively correlated with oxidative stress index in subjects with chronic hepatitis B (p = 0.008, r = 0.453). OSI, oxidative stress index; AU, arbitrary unit; ALT, alanine amino transferase.

TAR of chronic hepatitis B subjects was not significantly correlated with serum ALT level (p > 0.05). Serum ALT level were positively correlated with total peroxide level and OSI in subjects with chronic hepatitis B (p = 0.002, r = 0.519 and p = 0.008, r = 0.453, respectively) (Fig. 1 and 2).

There was no statistically significant correlation with ALT level, and TAR, total peroxide level and OSI in cirrhotic subjects or inactive HbsAg carriers or controls (all p > 0.05).

## Discussion

Normal cell functions and integrity of cell structures may be broken via considerable reactivity of ROS. The organism has enzymatic (e.g. superoxide dismutase, catalase, glutathione peroxidase) and non-enzymatic (e.g. vitamin C, vitamin E) antioxidant mechanisms that work as scavenger for this harmful ROS. Radical-scavenging antioxidants are consumed by the increased free radical activity associated with several conditions, and the total antioxidant response has been used to indirectly assess of free radical activity. The effects of various antioxidants in plasma are additive and the cooperation of antioxidants in human serum provides protection of the organism against attacks by free radicals [3,19]. Therefore, the measurement of TAR may reflect accurately the antioxidant status of the organism [3,12,15,20].

Oxidative stress can be defined as an increase in oxidants and/or a decrease in antioxidant capacity. Although determination of either oxidants or antioxidant components alone may give information about the oxidative stress, determination of oxidants along with antioxidants is more useful in this context. Therefore, oxidants and anti-oxidant capacity should be measured simultaneously to assess oxidative stress more exactly. In addition, the ratio of the total plasma peroxide level o TAR, regarded as OSI and an indicator of oxidative stress, reflects the redox balance between oxidation and anti-oxidation. Recently, it has been reported that OSI may reflect the oxidative status more accurately than TAR or total peroxide level alone [13,21].

Various methods have been developed for the measurement of total antioxidant status. However, there is not yet an accepted "gold standard" reference method [18,22-24], and decisions concerning standardization, and the terms and units used for the measurement of TAR have not yet been made [15]. This implies that this topic needs to be studied further [12]. The most widely used methods for TAR measurement are colorimetric, or involve either fluorescence or chemiluminescence [22,23,25]. However, the fluorescence and chemiluminescence methods need sophisticated techniques, are not appropriate for routine usage and not present in most routine clinical biochemistry laboratories.

In the present study, antioxidant capacity of subjects was determined using TAR, and oxidants and antioxidant capacity were determined simultaneously to determine oxidative stress. The novel method that used in the present study provides several major advantages in comparison with other currently available methods. It is simple and cheap, and can easily be fully automated. It is also reliable and sensitive, and does not interact with commonly occurring serum components such as bilirubin, serum lipids, and anticoagulants. Accurate measurements of TAR can be obtained as little as 10 minutes, making this assay eminently suitable for the clinical biochemistry laboratory [12].

The evidence of oxidative damage in human chronic viral hepatitis is accompanied by a significant rise of the plasma level of the fibrogenic cytokines TNFa and TGFb. In particular, the latter cytokine was shown increased already in plasma of patients with mild tissue inflammation in direct relation with the degree of tissue damage and fibrosis [5]. In addition, excess amounts of reactive species generated in inflamed tissues can cause injury to host cells and also induce DNA damage and mutations [26] and oxidative DNA damage has been suggested to play an important role in the development of HCC [6].

In several studies [7-9,11], increase in oxidative components or decrease in antioxidants or both have been reported in subjects with either acute or chronic HBV infection. Total antioxidant capacity in either acute or chronic HBV infection was measured in only in study of Irhsad et al [11]. The remaining was used individual antioxidants measurement to assess antioxidant response of the organism. At the same way, simultaneously measurement of the oxidants and antioxidant components of the plasma in CHB infection was performed in only at study of Demirdag et al [9].

The information in the literature about the antioxidant components in subjects with cirrhosis due to HBV infection is limited. Irhad et al [11] found that total antioxidant capacity of subjects either with cirrhosis due to HBV infection or other liver disease due to viral etiology is either comparable to or higher than control. To our knowledge, there is no information in the literature about the oxidants in subjects with cirrhosis due to HBV infection, and neither oxidants nor antioxidants in inactive HbsAg carrier subjects.

In order to reflect the true state of oxidative stress in the liver, measurement of lipid peroxidation markers and antioxidant components in hepatic tissue is more ideal than plasma. Nevertheless, ethical and practical considerations make this very difficult for research purposes. Liver biopsy caries a significant morbidity and even mortality risk and it is impossible to perform multiple tests with current techniques on very limited amounts of biopsy specimen that obtained in needle biopsy. Thus, in the present study, we have chosen to perform the measurement of oxidative stress markers in plasma samples. Indeed, in various disorders of the liver, increase in oxidants and/or decrease in antioxidants have been shown in both plasma and liver tissue samples [27,28].

It is well known that serum bilirubin has an antioxidant property [15]. Additionally, poor nutritional status caused modifications to the enzymatic antioxidant systems, with a lower ability to reduce oxidative compounds and a state of lipid peroxidation [29]. These two factors are frequently found in subjects with advanced stages of cirrhosis. Thus, in the present study, we included only the subjects with compensated Child A cirrhosis to evaluate the effects of cirrhosis due to HBV on oxidative status more accurately, and to exclude the effects of other additional factors.

In the present study, we found that TAR of CHB subjects was equivalent to inactive HBsAg carrier and controls. However, total peroxide level, a parameter of oxidative stress, and OSI was significantly higher in CHB subjects than inactive HBsAg carrier and controls. At the same way, there was a strict positive correlation between ALT level, and total peroxide level and OSI in CHB subjects, while no correlation between ALT level and TAR. Additionally, there was an inverse correlation between total peroxide level and OSI with TAR in cirrhotic subjects vs. inactive HBV carriers and controls.

Inactive HBsAg carrier and control subjects had comparable results in term of TAR, total lipid peroxide level and OSI. The lack of increase in TAR in the presence of increased oxidative components, and the strict correlation of ALT levels with total peroxide level and OSI are suggestive for the role of oxidative stress in the pathogenesis of CHB infection.

In the lightening of these findings, we concluded that oxidative stress may have a critical role in hepatic injury and is associated with the severity of disease and the replication status of virus in hepatitis B infection. The novel automated calorimetric assay is a useful, reliable, simple and easily applicable method in the assesment of the total plasma antioxidant response in various forms of hepatitis B virus infection.

## Abbreviations

ROS, reactive oxygen species; CHB, chronic hepatitis B infection; HCC, hepatocellular carcinoma; TAR, total antioxidant response; HBV, hepatitis B virus; HbsAg, hepatitis B surfage antigen; OSI, oxidative stress index; CHC, chronic hepatitis C; ALT, alanine amino transferase; HCV, hepatitis C virus; MEÝA, micro particle enzyme immunoassay; PCR, polymerase chain reaction.

## Competing interests

The author(s) declare that they have no competing interests.

## Authors' contributions

CB, FFB, OE, MA: Conception and design; CB, MH, HC: Analysis and interpretation of the data; CB, FFB, MH: Drafting of the article; CB, FFB, OE, MH, MA, HC: Critical revision of the article for important intellectual content; CB, FFB, OE, MH, MA, HC: final approval of the article; provision of study materials or patients; CB, FFB, MA; CB, MH: Statistical expertise; OE, MA, HC: Collection and assembly of data.

## Pre-publication history

The pre-publication history for this paper can be accessed here:


